# Biological Activity and Chemical Composition of Essential Oil from Leaves and Fruits of *Zanthoxylum mantaro* (J.F.Macbr.) J.F.Macbr

**DOI:** 10.3390/antibiotics14030216

**Published:** 2025-02-21

**Authors:** Vladimir Morocho, Odalis Eras, Teresa Rojas, Britany Jiménez, María Fernanda Roa, Luis Cartuche

**Affiliations:** 1 Departamento de Química, Universidad Técnica Particular de Loja (UTPL), Calle París s/n y Praga, Loja 110107, Ecuador; lecartuche@utpl.edu.ec; 2 Carrera de Bioquímica y Farmacia, Universidad Técnica Particular de Loja (UTPL), Calle París s/n y Praga, Loja 110107, Ecuador; goeras@utpl.edu.ec (O.E.); tdrojas1@utpl.edu.ec (T.R.); bajimenez20@utpl.edu.ec (B.J.); mfroa@utpl.edu.ec (M.F.R.)

**Keywords:** *Zanthoxylum mantaro*, α-thujone, β-thujone, germacrene D, antioxidant activity, anticholinesterase activity

## Abstract

**Objective**: In this study, the chemical composition and biological activities of the essential oils extracted from the leaves and fruits of *Zanthoxylum mantaro* were analyzed. **Methods**: The essential oils were obtained through hydrodistillation using a Clevenger-type apparatus. Chemical composition was determined by gas chromatography coupled with mass spectrometry (GC-MS) and gas chromatography with a flame ionization detector (GC-FID). The antimicrobial activity was evaluated against four Gram-positive bacteria, three Gram-negative bacteria, and two fungi using the broth microdilution method. Antioxidant activity was assessed using the ABTS (2,2′-azino-bis-3-ethylbenzothiazoline-6-sulfonic acid) and DPPH (2,2-diphenyl-1-picrylhydrazyl) radical scavenging assays. Additionally, the acetylcholinesterase inhibitory effect of the essential oils was measured by a spectrophotometric method. **Results and Conclusions**: A total of 23 compounds were identified in the essential oil from the fruits, while 47 compounds were found in the essential oil from the leaves. The major constituents of the fruit essential oil were α-thujone (39.85%), β-thujone (25.04%), sabinene (10.71%), and terpinen-4-ol (4.38%), whereas the main compounds in the leaf essential oil were germacrene D (21.75%), nerolidol (E) (12.39%), and pentadecanal (7.14%). The essential oil from the fruits exhibited antifungal activity against *Aspergillus niger* (ATCC 6275), with a minimum inhibitory concentration (MIC) of 1000 μg/mL. Both the fruit and leaf essential oils showed moderate antioxidant activity in the ABTS assay, with SC_50_ values of 274.14 ± 1.06 μg/mL and 2798.85 ± 15.69 μg/mL, respectively. Furthermore, the fruit essential oil demonstrated considerable acetylcholinesterase inhibitory activity with an IC_50_ value of 65.46 ± 1.01 μg/mL, while the leaf essential oil exhibited an IC_50_ value of 158.2 ± 1.02 μg/mL.

## 1. Introduction

Essential oils are complex mixtures of volatile compounds extracted from various aromatic plants, representing between 0.1% and 1% of their dry weight. The extraction of these hydrophobic compounds is primarily performed through hydrodistillation and steam distillation techniques, which allow the isolation of bioactive components. Various plant parts, such as leaves, flowers, roots, seeds, and even wood, can be used to obtain essential oils. These substances have a wide range of applications in the pharmaceutical, cosmetic, and food industries due to their biological and aromatic properties [1].

The Rutaceae family, commonly known as the citrus family, comprises approximately 160 genera and over 2000 species distributed predominantly in tropical and subtropical regions worldwide. Members of this family are known genera such as *Citrus*, *Zanthoxylum*, and *Ruta* [2,3]. This family is characterized by their aromatic properties and their economic and medicinal importance, which are attributed to the abundance of essential oils stored in secretory cavities within leaves, fruits, and bark. These compounds are not only responsible for their fragrance but are primarily due to the abundance of secondary metabolites such as alkaloids, flavonoids, and essential oils. These compounds exhibit a wide array of biological activities, including antimicrobial, antioxidant, anti-inflammatory, and anticancer properties [4,5].

The genus *Zanthoxylum*, commonly referred to as Sichuan pepper, comprises approximately 250 species distributed across Asia, Africa, and the Americas. This genus is particularly valued for its phytochemical diversity and ethnobotanical relevance, possessing alkaloids, flavonoids, terpenoids, and lignans, which exhibit a wide range of pharmacological properties. Notably, alkaloids such as skimmianine and nitidine have demonstrated antimicrobial, anti-inflammatory, and antimalarial activities, while essential oils from *Zanthoxylum* species are known for their potent insecticidal and antioxidant properties [6,7,8,9].

Ethnobotanical studies highlight the traditional uses of *Zanthoxylum* species in various cultures. For instance, the bark and seeds are commonly employed as spices, while decoctions are used in folk medicine for treating gastrointestinal disorders, rheumatism, and skin infections [10]. Additionally, the genus is widely used in several regions of Asia and Africa, and it is particularly mentioned in Thailand for spices or condiments for cooking, as reported by Okagu [11].

Pharmacological studies have validated several traditional uses of *Zanthoxylum*. The extracts and isolated compounds from species like *Zanthoxylum armatum*, *Zanthoxylum bungeanum*, and *Zanthoxylum americanum* exhibit antibacterial, antifungal, antinociceptive, and anti-inflammatory effects. Furthermore, the genus is being explored for its potential to develop natural insecticides and other eco-friendly agricultural products [12,13,14].

With the growing interest in natural products and their pharmaceutical applications, this preliminary study explores the chemical profile of *Zanthoxylum mantaro* essential oils from Ecuador, as well as their antimicrobial, antioxidant, and anticholinesterase (AChE) potential.

## 2. Results

### 2.1. Essential Oil Isolation

The essential oil (EO) of *Z. mantaro*, isolated through steam distillation, presented a characteristic citrus aroma and light-yellow color. The EO yielded 0.42% for the fruits and 0.72% for the leaves.

### 2.2. Chemical Composition of Essential Oil

Sixty-four compounds were identified by GC-MS and GC-FID in the essential oil samples from the leaves and fruits of *Z. mantaro* as depicted in Table 1.

**Figure 1:**
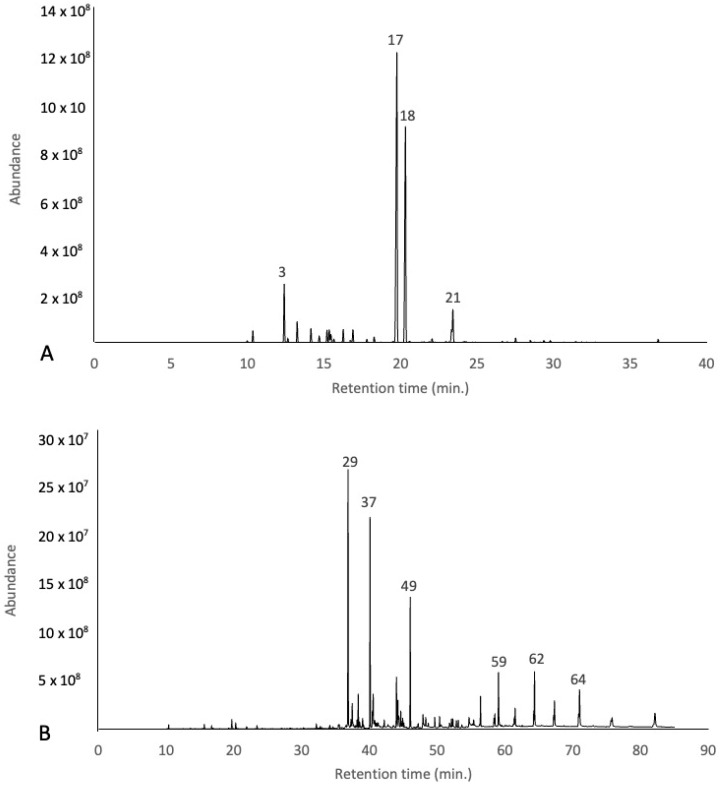
Representative GC-MS chromatogram of the essential oil from fruits (**A**) and leaves (**B**) of *Zanthoxylum mantaro*. The numbers above the peaks correspond to the numbering in Table 1.

A total of 23 compounds were identified for the fruit EOs, which represent 93.59% of the total chemical composition. The main compounds found were α-thujone (39.85%), β-thujone (25.04%), Sabinene (10.71%), and Terpinen-4-ol (4.38%) (Figure 1), and the EO was predominately composed of oxygenated monoterpenes (71.93%) followed by hydrocarbon monoterpene (20.37%).

In contrast, the EO from the leaves exhibited a greater chemical diversity, with 47 identified compounds, collectively constituting 90.86% of the total composition. This EO was rich in hydrocarbon sesquiterpenes (31.09%) and oxygenated sesquiterpenes (28.69%). The major components of this EO were Germacrene D (21.75%), (*E*)-Nerolidol (12.39%), Pentadecanal (7.14%), Phytol (3.77%), n-Pentacosane (3.37%), n-Tricosane (3.24%), and δ-Cadinene (2.69%) (Figure 1). Additionally, α-thujone (1.04%), β-thujone (0.74%), and Terpinen-4-ol (0,48%) were found as the minor compounds present in the essential oils (Figure 1).

### 2.3. Antimicrobial Activity of the Essential Oils of Z. mantaro

The antibacterial and antifungal activities of the essential oil from the leaves and fruits of *Z. mantaro* were determined using the broth microdilution method. Ampicillin, ciprofloxacin, and amphotericin B were used as positive controls and dimethylsulfoxide was used as the negative control. The maximum concentration tested was 4000 µg/mL. This study included four Gram-positive bacteria, three Gram-negative bacteria, and two fungi. The fruit essential oil exhibited a minimum inhibitory concentration (MIC) of 1000 µg/mL against *Aspergillus niger* (ATCC 6275). The results of the antimicrobial activity are presented in Table 2.

### 2.4. Scavenging Radical Capacity of Essential Oil

Two methods, ABTS (TEAC), and DPPH, were used to determine the antioxidant activity of the essential oil from the leaves and fruits of *Z. mantaro*. The scavenging capacity (SC_50_) was employed to report the results, where SC_50_ represents the concentration of the essential oil required to reduce the radical concentration by 50%. The fruit essential oil exhibited moderate to weak potential with an SC_50_ value of 274.14 ± 1.06 µg/mL with the ABTS method and 150.47 µg/mL. Trolox equivalent antioxidant capacity (TEAC) was calculated from ABTS data and expressed as µM Trolox/g EO. Both leaf and fruit EOs did not exert any effect on the DPPH radical at the maximum dose tested of 4000 µg/mL. Trolox was also used as a positive reference control (Table 3).

### 2.5. Anticholinesterase Activity

**Figure 2:**
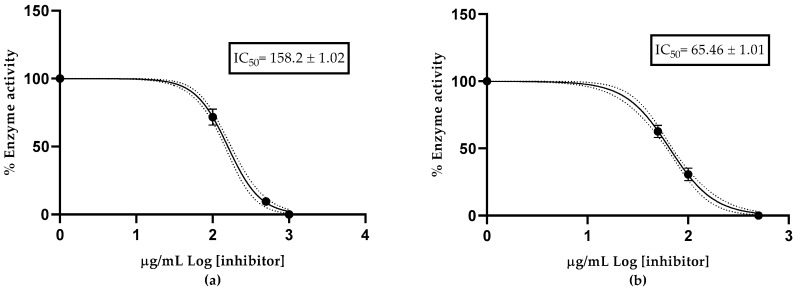
Half-maximum inhibitory concentration of *Z. mantaro* essential oil against acetylcholinesterase: (**a**) leaf EO and (**b**) fruit EOs.

The acetylcholinesterase inhibitory effect of the essential oil from the leaves and fruits of *Z. mantaro* is represented in the graph as the logarithm of the essential oil concentration vs. the normalized reaction response rate, enabling the calculation of the half-maximal inhibitory concentration (IC_50_) value (Figure 2). The essential oil from the leaves demonstrated an IC_50_ value of 158.2 ± 1.02 µg/mL, while the essential oil from the fruits exhibited an IC_50_ of 65.46 ± 1.01 µg/m. Donepezil, used as a positive control, exhibited an IC_50_ value of 12.40 ± 1.35 µg/mL.

## 3. Discussion

Essential oils have been isolated from various plant organs, with their relative percentages and compositions influenced by several factors. As revealed in this study, steam distillation of the fruits and leaves of *Zanthoxylum mantaro* showed significant variations in yield, with a higher yield observed in the essential oil (EO) from leaves (0.72%) compared to that from fruits (0.42%).

These yields are significantly lower than those reported for the fruit EO of *Z. lepidopteriphilum* (1.38%) [15] and *Z. leprieurii* (1.1%) [16]. However, they are closer to the reported yields for the leaf EO of *Z. armatum*, which range from 0.16% to 0.50% [17].

The higher concentration of secretory glands in the leaves of certain plant species has been widely documented as a key factor influencing variations in essential oil yields. For instance, the majority of *Zanthoxylum* spp. exhibits a significant presence of essential oil-producing glands in its leaves and pericarp, which enhances both the yield and quality of its oils [18].

Studies have demonstrated that the leaves of this species often produce a distinct chemical profile compared to its fruits, characterized by a higher abundance of terpenoid components, like linalool. These variations in secretory gland density and essential oil composition underscore the complex interplay between plant morphology and the production of secondary metabolites. Additionally, factors such as harvesting seasons and genetic differences among cultivars may further contribute to these variations [19,20,21].

The chemical analysis of the essential oils (EOs) from *Zanthoxylum mantaro* reveals a predominant composition of monoterpenes, with α-thujone (39.85%) and β-thujone (25.04%) as the main constituents in the fruits, whereas germacrene D (21.75%) and *E*-nerolidol (12.39%) are more prominent in the leaves. In a similar study, Morocho et al. [15] analyzed the EO of fruits from *Z*. *lepidopteriphilum* collected in Loja, Ecuador, and reported α-thujone (70.26%) and β-thujone (10.78%) as the major compounds, findings that are consistent with our results. The essential oil from *Z. lepidopteriphilum* also showed a predominance of monoterpenes (76.63%), with oxygenated monoterpenes (90.21%) dominating over monoterpene hydrocarbons (8.50%).

In contrast, the chemical composition of *Z. armatum*, collected under distinct geographic and climatic conditions in China, showed a different profile, with linalool (55.65%) and d-limonene (39.97%) as the main components [22]. Despite these differences, both species share the presence of (-)-terpinen-4-ol, although at varying concentrations. This suggests possible similarities in their biosynthetic pathways within the *Zanthoxylum* genus [23].

Studies on *Z. acanthopodium* conducted in China and Myanmar revealed variations in its chemical composition, with terpinen-4-ol (43.35%) and β-myrcene (26.65%) as the predominant compounds in the analyzed essential oils [24]. Similarly, the essential oil of *Z. bungeanum*, a widely utilized spice in Asian cuisine, is dominated by terpineol-4-ol (13.13%), (-)-β-pinene (11.17%), γ-terpinene (9.45%), terpinyl acetate (9.36%), and α-terpineol (5.40%) [25]. These differences may be attributed to factors such as genetic variability, environmental conditions, and extraction methods [26].

In *Z. piperitum*, the major components include linalol (18%), Geranyl actetate (15.3%), and cryptone (8.5%); meanwhile, for *Z. schinifolium* B phellandrene (22.54%), Citronellal (16.48%) and Geranyl acetate (11.39%) were reported as the main compounds. These EOs were reported as remarkable antimicrobial foodborne pathogens as revealed in this study, where MIC values obtained were 1.25, 2.5, and 1.25 µg/mL against *Bacillus cereus*, *Staphylococcus aureus,* and *Vibrio parahaemolyticus,* respectively [27].

Additionally, the essential oils from *Zanthoxylum alatum* are characterized by a high content of linalool (56.10%) and methyl cinnamate (19.73%) as major components. These compounds have demonstrated practical efficacy as plant-based antimicrobials, particularly in the post-harvest preservation of *Piper nigrum* L. fruits [28].

Regarding antimicrobial activity, the essential oil (EO) from the fruits of *Zanthoxylum mantaro* exhibited moderate activity against the fungus *Aspergillus niger* (ATCC ^®^ 6275), with a minimum inhibitory concentration (MIC) value of 1000 µg/mL based on the classification proposed by Van Vuuren [29].

There are no specific reports on the antifungal properties of the main chemical constituents of the fruit EO, such as α-thujone and β-thujone, against *Aspergillus niger*. However, Teker et al. [30] demonstrated that α-thujone exhibits a strong inhibitory profile against *Fusarium graminearum* by inducing apoptosis and oxidative stress.

The moderate antifungal activity of the fruit essential oil (EO) may be attributed to its monoterpene content, particularly α-thujone and sabinene. α-Thujone is known for its antimicrobial properties, while sabinene, a bicyclic unsaturated monoterpene widely found in plants, is a key component of various essential oils used in the perfume, flavor, and pharmaceutical industries. Sabinene has been reported to exhibit antifungal and anti-inflammatory properties, which may contribute to the observed activity [31].

The essential oil (EO) from the fruits exhibited moderate antioxidant activity as determined by the ABTS method. However, neither the fruit nor the leaf EO showed antioxidant activity using the DPPH method. The lack of activity in the DPPH assay may be attributed to the inability of terpene compounds to donate hydrogen atoms, which is a critical mechanism evaluated by this method. In contrast, the ABTS method is considered more suitable for assessing the antioxidant activity of essential oils [32,33].

A mechanistic approach can be explained as follows. The ABTS radical cation is soluble in both hydrophilic (water-based) and lipophilic (oil-based) environments, making it versatile for assessing antioxidants in diverse systems, including essential oils; meanwhile, the DPPH radical is soluble primarily in methanol and ethanol, which may not effectively solubilize all components of essential oils, as explained by [34]. Additionally, the abstraction of hydrogen from the sample by the DPPH radical is marginal because it occurs very slowly and depends on the hydrogen bond-accepting solvent. Methanol, for instance, is a strong hydrogen bond-accepting solvent; therefore, the hydrogen-abstracting reaction occurs very slowly [35].

Previous studies have shown that *Zanthoxylum* species exhibit significant antioxidant effects, which can be utilized for the prevention and management of oxidative stress-related conditions [11,36,37].

The acetylcholinesterase (AChE) inhibitory activity of the essential oils (EOs) from the leaves and fruits of *Zanthoxylum mantaro* has not been previously reported. The oils demonstrated a promising inhibitory profile, with IC_50_ values of 158.2 ± 1.02 µg/mL for the leaf EO and 65.46 ± 1.01 µg/mL for the fruit EO.

Chemical constituents of the *Zanthoxylum* genus have shown notable effects on the central nervous system, with potential therapeutic applications for neurodegenerative diseases, such as Alzheimer’s [38]. Essential oils and their components have been extensively documented for their neuroprotective properties, and they can be used as potential remedies for Alzheimer’s disease; they also offer advantages as additives and packaging materials in the food industry, as well as in perfumes and cosmetics [39].

Although *Zanthoxylum mantaro* has not been extensively studied, *Zanthoxylum* species are known to contain a diverse array of chemical compounds, including alkaloids and coumarins from crude organic extracts and terpene-like compounds from essential oils. These compounds exhibit a wide range of biological activities, such as larvicidal, anti-inflammatory, analgesic, antinociceptive, antioxidant, antibiotic, hepatoprotective, antiplasmodial, cytotoxic, antiproliferative, anthelmintic, antifungal, and antiviral properties, as reported by Negi et al. [40].

A new alkaloid, schifoline, along with bergapten, umbelliferone, and skimmianine, was isolated from *Zanthoxylum schinifolium*, a traditional Chinese medicinal plant known as “Qinghuajiao”, as reported by Liu [41]. More recently, Yang et al. [42] discovered that skimmianine, a furoquinoline alkaloid, exhibits a notable AChE inhibitory profile with an IC_50_ value of 8.06 µg/mL, further highlighting the importance of investigating the chemistry of this genus.

In a study on the essential oil composition of *Zanthoxylum* species, *Z. piperitum* and *Z*. *armatum* were analyzed for their potential insecticidal activity. Despite exhibiting notable AChE inhibitory effects, no direct correlation with fly toxicity was observed. However, based on EO composition and AChE tests, strong AChE inhibition was attributed to citronellyl acetate, α-pinene, thymol, carvacrol, and α-terpineol (1.20–2.73 mM) [43], reinforcing the idea that essential oils from *Zanthoxylum* spp. could serve as potential sources of AChE inhibitors.

The majority of terpenes have shown strong inhibitory activity against AChE or Butyrylcholinesterase (BuChE), as summarized by Lai Shi Min [44]. Notably, abietane-type diterpenes, triterpenoids, limonoids, and sesquiterpenoids have exhibited inhibition of AChE or BuChE within the micromolar range.

The essential oil (EO) from *Z. mantaro* leaves exhibited high concentrations of germacrene D, followed by nerolidol and pentadecanal, which are likely responsible for the observed anticholinesterase activity. Although the literature on germacrene D as an AChE inhibitor is scarce, Kang et al. [45] reported that cis-nerolidol exhibited mild AChE inhibition (58% at 100 mg/mL) in *Bursaphelenchus xylophilus*, while Szwajgier and Baranowska-Wójcik [46] first documented BuChE inhibition by nerolidol at approximately 30.8% at 0.84 mmol/L. These findings may explain the moderate inhibitory effect observed in our study.

The higher inhibitory potential of the fruit EO compared to the leaf EO could be attributed to the presence of sabinene, which, according to Menichini, exhibited significant AChE inhibition with an IC_50_ value of 176.5 µg/mL [47]. While no literature exists on the cholinesterase inhibitory potential of α- and β-thujone, the main components of *Z*. *mantaro* fruit EO, a study by Politeo et al. [48] on six *Artemisia* species reported that *A. verlotiorum* EO (46.5% β-thujone) and *A. vulgaris* EO (40.3% α-thujone) exhibited AChE inhibition of 34.3% and 54.4% at 1 mg/mL, respectively. These findings suggest that the major compounds in the fruit EO may exert their effect through an additive or synergistic mechanism.

Phytochemical chromatographic studies could be conducted to isolate the main chemical constituents of both EOs, given their high relative abundance in the EO composition. Although the low yield may pose a limitation, such studies could help elucidate the precise mechanism of AChE inhibition. Insights from this research may contribute to the development of pharmaceutical adjuvants for Alzheimer’s disease treatment.

## 4. Materials and Methods

### 4.1. General Information

The chemical analysis of *Z. mantaro* essential oil (EO) was performed using a gas chromatograph (Trace 1310) coupled to a single quadrupole mass spectrometry detector, model ISQ 7000, and a common flame ionization detector (FID) (both from Thermo Fisher Scientific, Waltham, MA, USA). The qualitative and quantitative analyses were conducted using a non-polar stationary phase capillary column, TR-5MS (30 m long, 0.25 mm internal diameter, and 0.25 μm film thickness), purchased from Thermo Fisher Scientific, Waltham, MA, USA. For all analyses, GC purity-grade helium (Indura, Guayaquil, Ecuador) was used as the carrier gas. The standard aliphatic hydrocarbons were purchased from ChemService (West Chester, PA, USA). Dichloromethane, anhydrous sodium sulfate, 2,2′-azinobis-3-ethylbenzothiazoline-6-sulfonic acid (ABTS), 2,2-diphenyl-1-picrylhydryl (DPPH), 5,5′-dithiobis (2-nitrobenzoic acid) (DTNB), acetylcholinesterase (AChE), acetylthiocholine (AcSCh), dimethylsulfoxide (DMSO), donepezil, methanol (MeOH), magnesium chloride hexahydrate, phosphate-buffered saline (PBS), and tris hydrochloride (tris-HCl) were purchased from Sigma-Aldrich (San Luis, MO, USA). Mueller–Hinton broth, Mueller–Hinton II broth, and fluid thioglycollate medium were purchased from DIPCO (Quito, Ecuador).

### 4.2. Plant Material

The leaves and fruits of *Z*. *mantaro* were collected in June 2024 at Guachanama hill, located in Loja Province in Southern Ecuador (4°05′58″ S y 79°57′08″ W, 2110 m a.s.l.). The collection of plant material was carried out under authorization from the Ministry of Environment, Water, and Ecological Transition of Ecuador (MAATE) with permit code MAE-DBN-2016-048 of the Ecuadorian Government. The taxonomical identification was performed by Nixon Cumbicus, a botanist at the Herbarium UTPL. A specimen sample has been deposited in the UTPL Herbarium (HUTPL) with the voucher code PPN-RU-010.

### 4.3. Extraction of Essential Oil

The fruits and leaves were steam distilled using a stainless-steel Clevenger-type apparatus immediately after harvesting for two hours. The process was performed in triplicate with 100 g of plant material per distillation in an open system at 0.775 atm, with water boiling at 92 °C (2100 m a.s.l.). The essential oils obtained were dried over anhydrous sodium sulfate, stored in vials at 4 °C, and protected from light until further chemical and biological analyses.

### 4.4. Identification and Quantification of Essential Oil

#### 4.4.1. Sample Preparation

Both leaf and fruit EOs from *Z. mantaro* were dissolved at a 1:10 ratio in methylene chloride. A total of 10 µL of the sample was dissolved in 990 µL of methylene chloride HPLC grade from Sigma (St. Louis, MO, USA) (990 µL).

#### 4.4.2. Qualitative Analysis (GC-MC) of the EOs

The composition analysis was conducted on a Thermo Fisher Scientific Trace 1310 Gas Chromatograph (GC) equipped with a Thermo Scientific AI/AS 1300 liquid sampling automation system and an ISQ 7000 Single Quadrupole Mass Spectrometer (Thermo Fisher Scientific, Waltham, MA, USA). A non-polar column TR5-ms (0.25 mm × 30 m with a thickness of 0.25 µm) with 5%-Phenyl-Polysilphenylene-Siloxane (Thermo Fisher Scientific, Waltham, MA, USA) was used as the stationary phase.

The mass spectrometer was operated in electron impact ionization mode at 70 eV, with a mass range of 40–400 *m*/*z* in full scan mode. The ion source temperature was set at 220 °C and the transfer line at 230 °C.

The initial oven temperature was held at 50 °C for 5 min with a ramp of 3 °C/min until reaching 180 °C and a second ramp of 15 °C/min until finally reaching the temperature of 230 °C. The injector temperature was 250 °C, and a split ratio of 40:1 was adjusted for sample injection. Helium was used as a carrier gas at 1 mL/min in constant flow mode.

#### 4.4.3. Quantitative Analysis (GC-FID) of the EOs

For the quantitative analysis, the same TR-5ms column mentioned above was used. The analysis was performed on the same equipment coupling a flame ionization detector (FID). Injection and operation conditions were the same as performed for GC-MS analysis.

#### 4.4.4. Identification and Quantification of Compounds

Volatile compounds were identified by comparing their linear retention indices (LRIs) and mass spectra with published data [49]. LRI values were determined using Equation (1) derived from the Van Den Dool and Krats method [50] based on homologous standard aliphatic hydrocarbons (TPH-6RPM, Chem Service, C9–C24) injected under the same conditions as the oils.

(1) $$LRI=100C+100\frac{\left(RTx-RTn\right)}{\left(RTN-RTn\right)}$$

where C represents the carbon number of aliphatic hydrocarbons (C9 to C25) that elute either before or after the compound of interest. RTx is the retention time of the compound of interest, RTn is the retention time of the aliphatic hydrocarbons that elute before the compound of interest, and RTN is the retention time of the hydrocarbons that elute after the compound of interest.

Volatile compounds were quantified by integrating GC-FID peak areas, and the percentage composition of the oils was determined using the normalization method. Calculations were based on three injections per oil without applying correction factors.

### 4.5. Antimicrobial Activity

The antimicrobial activity of essential oils extracted from the leaves and fruits was evaluated using the broth microdilution method, following the protocol described by Cartuche et al. [51]. The assay included a range of American-Type Culture Collection (ATCC) strains representing common human pathogens. Gram-positive bacteria tested were *Enterococcus faecalis* (ATCC 19433), *Enterococcus faecium* (ATCC 27270), and *Staphylococcus aureus* (ATCC 25923). Gram-negative bacteria included *Escherichia coli* O157:H7 (ATCC 43888), *Pseudomonas aeruginosa* (ATCC 10145), *Salmonella enterica* (ATCC 14028), and *Campylobacter jejuni* (ATCC 33560). Additionally, *Candida albicans* (ATCC 10231) and *Aspergillus niger* (ATCC 6275) were evaluated for antifungal activity. Minimum inhibitory concentrations (MICs) were determined to quantify the antimicrobial potential. Dimethyl sulfoxide (DMSO) served as the negative control, while ampicillin, ciprofloxacin, and amphotericin B were used as positive controls for Gram-positive bacteria, Gram-negative bacteria, and fungi, respectively. Broth the microdilution method and the twofold serial dilution system were employed to obtain concentrations ranging from 4000 to 31.25 µg/mL of EOs. The final inoculum concentrations were set at 5 × 10^5^ CFU/mL for bacteria, 2.5 × 10^5^ CFU/mL for yeast, and 5 × 10^4^ spores/mL for sporulated fungi.

### 4.6. Radical Scavenging Capacity

The antioxidant activity of the essential oils extracted from the leaves and fruits was evaluated using the DPPH and ABTS radical scavenging assays, following the methodology described by Cartuche et al. [51]. In the DPPH assay, a working solution of 2,2-diphenyl-1-picrylhydrazyl (DPPH) was prepared by dissolving 24 mg of DPPH in 100 mL of methanol and then adjusted to an absorbance of 1.1 ± 0.01 at 515 nm using an EPOCH 2 microplate reader (BIOTEK, Winooski, VT, USA). Essential oils were dissolved in methanol (10 mg/mL), and a twofold serial dilution system was used to reach final concentrations ranging from 4000 to 31.25 µg/mL Each solution was reacted with the DPPH-adjusted solution in 96-microwell plates and monitored for 60 min. For the ABTS assay, the radical cation ABTS was generated by mixing ABTS (7.4 µM) and potassium persulfate (2.6 µM), followed by stirring for 12 h. The ABTS working solution was adjusted with methanol to an absorbance of 1.1 ± 0.02 at 734 nm and reacted with essential oils at the same concentrations described above. Both assays were performed in triplicate, and the results were expressed as SC_50_, the concentration required to achieve 50% radical scavenging activity. Trolox served as a positive control, while methanol was used as a negative control. The UV absorbance was measured at 515 nm for DPPH and 734 nm for ABTS using an EPOCH 2 microplate reader.

### 4.7. Cholinesterase Assay

The acetylcholinesterase (AChE) inhibitory activity of the leaves and fruits of essential oils (EOs) from *Z. mantaro* was evaluated in vitro using the spectrophotometric method developed by Ellman et al. [52], with modifications proposed by Andrade et al. [53]. The assay utilized the AChE enzyme from *Electrophorus electricus* (Sigma Aldrich, San Luis, MO, USA), and the reaction was monitored at 405 nm using an EPOCH 2 microplate reader (BioTek, Winooski, VT, USA). Reaction mixtures included tris buffer pH 8.0, acetylthiocholine (ATCh), and 5,5′-dithiobis-(2-nitrobenzoic acid) (DTNB), with EO concentrations ranging from 10 to 1000 μg/mL prepared by serial dilution in methanol (MeOH). A pre-incubation period of 3 min at 25 °C with continuous shaking preceded the addition of 0.5 U/mL AChE to initiate the reaction, which was monitored for 60 min. IC_50_ values, representing the EO concentration required for 50% enzyme inhibition, were calculated using non-linear regression analysis (GraphPad Prism 8.0.1, GraphPad, San Diego, CA, USA). Donepezil hydrochloride was used as a positive control, exhibiting an IC_50_ value of 12.40 ± 1.35 nM, while methanol served as a negative control. Absorbance adjustments accounted for spontaneous ATCh hydrolysis to ensure accuracy.

## 5. Conclusions

The chemical composition and biological activity of essential oil from leaves and fruits of *Zanthoxylum mantaro* were determined for the first time; 47 compounds were identified in the leaf essential oil, of which Germacrene D (21.75%), *E*-Nerolidol (12.39%), and Pentadecanal (7.14%) were the most representative, and 23 were identified in the fruit essential oil. The main compounds were α-thujone (39.85%), β-thujone (25.04%), Sabinene (10.71%), and Terpinen-4-ol (4.38%). The fruit essential oil was characterized by a predominance of oxygenated monoterpenes constituting around 71.93% of total oil. Biologically, it exhibited moderate anticholinesterase and antioxidant activity. *Zanthoxylum mantaro* demonstrates significant potential as a source of bioactive compounds, making it highly promising for applications in the pharmaceutical, cosmetic, and food industries. Its notable antimicrobial, antioxidant, and neuroprotective properties highlight its therapeutic value. Further research on its chemical composition and biological activities will provide deeper insights into its mechanisms of action and broaden its potential as a versatile therapeutic agent.

## Figures and Tables

**Table 1:** Chemical composition of the EO from *Zanthoxylum mantaro* fruits and leaves obtained through the DB5-MS non-polar capillary column.

N°	Compound ^a^	CLRI	LLRI	Leaves		Fruits		CF
				%	SD	%	SD	
1	α-Thujene	926	924	0.45	0.022	0.32	0.004	C_10_H_16_
2	α-Pinene	933	932	-	-	0.64	0.007	C_10_H_16_
3	Sabinene	975	969	-	-	10.71	0.112	C_10_H_16_
4	β-Pinene	979	974	0.15	0.06	0.60	0.007	C_10_H_16_
5	Myrcene	993	988	-	-	1.86	0.020	C_10_H_16_
6	α-Phellandrene	1009	1002	-	-	0.93	0.010	C_10_H_16_
7	α-Terpinene	1020	1014	-	-	0.51	0.006	C_10_H_16_
8	ο-Cymene	1030	1022	-	-	1.24	0.010	C_10_H_14_
9	Limonene	1033	1024	-	-	0.97	0.010	C_10_H_16_
10	β-Phellandrene	1034	1025	-	-	0.62	0.006	C_10_H_16_
11	1,8-Cineole	1037	1026	0.48	0.02	0.32	0.005	C_10_H_18_O
12	trans-Ocimene	1052	1044	-	-	0.98	0.010	C_10_H_16_
13	γ-Terpinene	1063	1054	-	-	0.99	0.010	C_10_H_16_
14	trans-4-Thujanol	1079	1065	-	-	0.64	0.006	C_10_H_18_O
15	*p*-Mentha-2,4(8)-diene	1089	1085	-	-	0.31	0.003	C_10_H_18_O
16	trans-Pinene hydrate	1111	1119	-	-	0.41	0.012	C_10_H_18_O
17	α-thujone	1117	1101	1.04	0.05	39.85	0.363	C_10_H_16_O
18	β-thujone	1128	1112	0.74	0.04	25.04	0.302	C_10_H_16_O
19	trans-*p*-Menth-2-en-1-ol	1134	1136	-	-	0.27	0.004	C_10_H_18_O
20	trans-Pinocamphone	1187	1169	-	-	0.72	0.012	C_10_H_18_O
21	Terpinen-4-ol	1190	1175	0.48	0.02	4.38	0.040	C_10_H_18_O
22	Isothujyl acetate	1277	1266	-	-	0.76	0.007	C_12_H_20_O_2_
23	trans-Sabinyl acetate	1297	1297	-	-	0.53	0.007	C_12_H_20_O_2_
24	Isoledene	1381	1374	0.60	0.019	-	-	C_15_H_24_
25	β-Copaene	1437	1430	0.56	0.011	-	-	C_15_H_24_
26	α-neo-Clovene	1456	1452	0.44	0.008	-	-	C_15_H_24_
27	Geranyl acetone	1459	1453	0.40	0.010	-	-	C_13_H_22_O
28	γ-Muurolene	1483	1478	0.24	0.004	-	-	C_15_H_24_
29	Germacrene D	1489	1480	21.75	0.279	-	-	C_15_H_24_
30	n-Pentadecane	1500	1500	1.53	0.013	-	-	C_15_H_32_
31	Isolepidozene	1504	1480	2.00	0.043	-	-	C_15_H_24_
32	δ-Amorphene	1510	1511	0.27	0.005	-	-	C_15_H_24_
33	γ-Cadinene	1522	1513	0.31	0.210	-	-	C_15_H_24_
34	δ-Cadinene	1526	1522	2.69	0.032	-	-	C_15_H_24_
35	Zonarene	1532	1528	0.56	0.068	-	-	C_15_H_24_
36	α-Cadinene	1546	1537	0.14	0.122	-	-	C_15_H_24_
37	(*E*)-Nerolidol	1570	1561	12.39	0.083	-	-	C_15_H_26_O
38	(*Z*)-dihydro-Apofarnesol	1578	1571	2.63	0.110	-	-	C_15_H_28_O
39	Dodecanoic acid	1581	1580	1.96	0.009	-	-	C_12_H_24_O_2_
40	(*Z*)-3-Hexen-1-ol, benzoate	1588	1580	0.53	0.004	-	-	C_13_H_16_O_2_
41	Isoaromadendrene epoxide	1597	1594	0.75	0.006	-	-	C_15_H_24_O_2_
42	Tetradecanal	1622	1615	0.52	0.007	-	-	C_14_H_28_O
43	Junenol	1638	1618	0.39	0.214	-	-	C_15_H_26_O
44	(2*Z*,6*Z*)-Farnesal	1671	1684	2.91	0.021	-	-	C_15_H_26_O
45	9,12,15-Octadecatrienal	1676	1676	2.10	0.034	-	-	C_18_H_30_O
46	n-Tetradecanol	1679	1671	1.49	0.045	-	-	C_14_H_30_O
47	13-Methyltetradecanal	1688	1680.3	1.24	0.062	-	-	C_15_H_30_O
48	n-Heptadecane	1700	1700	0.25	0.020	-	-	C_17_H_36_
49	Pentadecanal-	1726	1717	7.14	0.047	-	-	C_15_H_30_O
50	Isobicyclogermacrenal	1758	1733	1.23	0.005	-	-	C_15_H_22_O
51	Tetradecanoic acid	1778	1768	1.21	0.007	-	-	C_14_H_28_O
52	2-Pentadecanone, 6,10,14-trimethyl-	1848	1846.7	0.71	0.002	-	-	C1_8_H_36_O
53	n-Nonadecane	1899	1900	0.42	0.220	-	-	C_19_H_40_
54	(5*E*,9*E*)-Farnesyl acetone	1921	1913	0.53	0.004	-	-	C_18_H_28_O
55	n-Eicosane	1999	2000	0.33	0.008	-	-	C_20_H_42_
56	(*E*,*E*)-Geranyl linalool	2031	2026	2.11	0.024	-	-	C_20_H_34_O
57	n-Octadecanol	2095	2077	0.62	0.041	-	-	C_18_H_38_O
58	n-Heneicosane	2099	2100	1.02	0.048	-	-	C_21_H_44_
59	Phytol	2117	2116	3.77	0.006	-	-	C_20_H_40_O
60	1-Docosene	2195	2189	0.53	0.006	-	-	C_22_H_44_
61	n-Docosane	2199	2200	0.78	0.007	-	-	C_22_H_46_
62	n-Tricosane	2300	2300	3.24	0.121	-	-	C_23_H_48_
63	n-Tetracosane	2404	2400	1.86	0.058	-	-	C_24_H_50_
64	n-Pentacosane	2535	2500	3.37	0.013	-	-	C_25_H_22_
	Monoterpenes hydrocarbons				0.60	20.37		
	Oxygenated monoterpenes				2.74	71.93		
	Sesquiterpenes hydrocarbons				31.09	0.00		
	Oxigenated sesquiterpenes				28.69	0.00		
	Others				27.74	1.29		
	Total identified				90.86	93.59		

^a^ Compounds ordered according to the elution time; CLRI: calculated retention index; LLRI: retention index from reference; SD: standard deviation; CF: chemical formula.

**Table 2:** Antimicrobial activity of the EO from the leaves and fruits of *Z. mantaro*.

Microorganism	Leaves	Fruits	Positive Control ^a^
	MIC (μg/mL)		
Gram-positive cocci			
*Enterococcus faecalis* (ATCC ^®^ 19433)	4000	4000	0.7812
*Enterococcus faecium* (ATCC ^®^ 27270)	4000	4000	<0.3906
*Staphylococcus aureus* (ATCC ^®^ 25923)	-	-	<0.3906
Gram-negative bacilli			
*Escherichia coli O157:H7* (ATCC ^®^ 43888)	-	-	1.5625
*Pseudomonas aeruginosa* (ATCC ^®^ 10145)	-	-	<0.3906
*Salmonella enterica* subs enterica serovar Thypimurium WDCM 00031, derived (ATCC ^®^ 14028)	-	-	<0.3906
Gram-positive bacilli			
*Lysteria monocytogenes* (ATTC ^®^ 19115)	-	-	1.5625
Yeasts and sporulated fungi			
*Candida albicans* (ATTC ^®^ 10231)	-	-	<0.098
*Aspergillus niger* (ATCC ^®^ 6275)	4000	1000	<0.098

^a^ Ampicillin for *Enterococcus faecalis*, *Enterococcus faecium*, and *Staphylococcus aureus*; ciprofloxacin for *Escherichia coli*, *Pseudomonas aeruginosa*, *Salmonella enterica*, and *Listeria monocytogenes*; amphotericin B for *Candida albicans* and *Aspergillus niger*.

**Table 3:** Half-scavenging capacity (SC_50_) of essential oil from the leaves and fruits of *Z. mantaro*.

Sample	ABTS	TEAC	DPPH
	SC_50_ (µg/mL—µM *) ± SD		
*Z.mantaro leaves* EO	2798.85 ± 15.69	10.96 ± 1.64	-
*Z. mantaro fruit* EO	274.14 ± 1.06	150.47 ± 27.37	-
Trolox *	29.09 ± 1.05		35.54 ± 1.04

* Trolox was used as a positive reference, and its values are given in µM; (-) No effect at the maximum dose tested.

## Data Availability

Raw data availability statements are available from the authors (V.M. and L.C.).
